# Chemical and genetic discrimination of commercial Guangchenpi (*Citrus reticulata* ‘Chachi’) by using UPLC-QTOF-MS/MS based metabolomics and DNA barcoding approaches†

**DOI:** 10.1039/c9ra03740c

**Published:** 2019-07-29

**Authors:** Peng Wang, Jing Zhang, Yating Zhang, He Su, Xiaohui Qiu, Lu Gong, Juan Huang, Junqi Bai, Zhihai Huang, Wen Xu

**Affiliations:** The Second Clinical College of Guangzhou University of Chinese Medicine Guangzhou China freexuwen@163.com; Guangdong Provincial Key Laboratory of Clinical Research on Traditional Chinese Medicine Syndrome, Guangdong Provincial Hospital of Traditional Chinese Medicine Guangzhou China zhhuang7308@163.com

## Abstract

CRP (Citri Reticulatae Pericarpium), a famous traditional Chinese medicine, has also been extensively used in foods and condiments in dietary practice for centuries. According to the Chinese Pharmacopeia (2015 edition) it contains two subtypes, Guangchenpi (GCP) and Chenpi (CP). GCP exclusively originates from the pericarp of *Citrus reticulata* ‘Chachi’ cultivar and it's generally believed that GCP has superior qualities compared with the other main cultivars (CP). In the present study, an integrated approach combining LC-QTOF MS-based untargeted metabolomics analysis and DNA barcoding molecular identification was conducted to study the genetic diversity and chemical differences between GCP and CP. A validated UPLC-QTOF MS metabolomics method was established to identify markers by using PCA and OPLS-DA models. 34 identified metabolites could be used as chemical markers to distinguish effectively between the two subtypes. Among them polymethoxyflavones (PMF) such as hexamethoxyflavone (nobiletin and natsudaidain), pentamethoxyflavone (tangeretin and sinensetin), and tetramethoxyflavone are the most influential markers. Support vector machines were employed to classify all the samples and these markers showed good prediction accuracy (100%). The results of DNA barcoding showed that the secondary structure of the ITS2 sequences were significantly different among GCP and other three cultivars. The study indicated the integrated method could be a powerful and reliable analytical tool for differentiating GCP from CP.

## Abstract 2

An integrated method, combining DNA barcoding with LC-MS metabolomics analysis, is a powerful and reliable way for differentiating *Citrus reticulata* ‘Chachi’ (GCP) from other *Citrus* cultivars.

## Graphical abstract

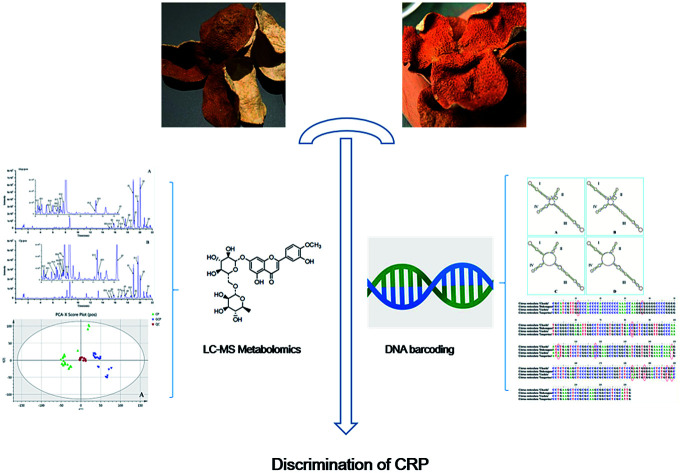

## 1. Introduction

Citri Reticulate Pericarpium (CRP), the dried matured tangerine pericarp, is one of the most well-known Chinese medicinal materials which has long been extensively used for health purposes. According to traditional Chinese Medicine theory, CRP has been used as a Qi-regulating herb to treat nausea, vomiting, indigestion, anepithymia, diarrhea, coughs, expectoration, and so on.^1^ Besides a medical purpose, CRP has been frequently used in foods, drinks, snacks, health-care products and condiments in China and Southeast Asia.^2^ In recent years, many researchers have focused on the pharmacological actions of CRP, such as beneficial effects on the gastrointestinal and respiratory systems, antioxidant, anti-inflammatory, and anti-cancer activities, as well as protective effects on the liver and nervous system.^3–6^

CRP is divided into two subtypes, Guangchenpi (GCP) and Chenpi (CP), as stated unambiguously in the current version of Chinese Pharmacopeia.^7^ Generally, GCP is defined as pericarp of a specific cultivar of *Citrus reticulata*, namely *Citrus reticulata* ‘Chachi’, while other main cultivars refer to CP, including *Citrus reticulata* ‘Dahongpao’, *Citrus reticulata* ‘Unshiu’, *Citrus reticulata* ‘Tangerina’, and so on. As a rule, *Citrus reticulata* ‘Chachi’, cultivated and harvested in a limited area, Xinhui county of Guangdong Province in China, is regarded as the genuine source of GCP,^8^ while CP is widely distributed cultivated within China. In spite of moderate differences in morphological traits between CP and GCP, their commercial products are frequently confused as some morphological traits have been lost after processing. There is no systemic method to distinguish GCP and CP, however, the current method relies on visual and morphological evaluation by artificial experience. According to recent studies, chemical composition of GCP and CP often vary among varieties, which could affect their quality and pharmacological effects.^9,10^ Thus, it is imperative to investigate their genetic and chemical differences for discrimination and quality control of these similar subtypes.

DNA barcoding methods have been used to overcome the limitations of morphological identification in plant discrimination based on the DNA sequences, such as the ITS region of nrDNA, and chloroplast DNA (cpDNA) rbcL, psbA-trnH, and matK.^11,12^ Previous studies have demonstrated the ability of DNA barcoding to identify *Citrus* plants.^13^ The internal transcribed spacer 2 (ITS2) which is part of the eukaryotic nuclear rDNA cistron and lies between the 5.8S and 28S rDNA, as a novel DNA barcode for identifying medicinal plant species.^14^ Compared with other sequences, the advantage of ITS2 is the standard sequence that can be obtained through the website annotation (http://its2.bioapps.biozentrum.uni-wuerzburg.de/)^15^ and predicted secondary structure of ITS2 from the database helps us to learn more about the genetic information of species for identification. A previous study has shown that there are differences in the secondary structures of ITS2 sequence between *Citrus reticulata* ‘Chachi’ and other two cultivars.^16^

Recently developed metabolomics analysis method is regarded as a powerful technology that could systematically and comprehensively investigate the metabolic profiles in different samples, which enable us to reveal the differences of chemical features among the closely related species of traditional Chinese Medicines.^9,10^ Previously studies have successfully applied GC-MS and/or LC-MS based metabolomics analysis for discriminating the *Citrus* genus products such as the *Citrus* peel,^9^ fruit,^17^ and/or fruit juice.^18^ However, basing on a synthetic strategy combing metabolomics analysis and DNA barcoding methods could be a more practical solution to make integrated evaluation on their cultivar differentiation.

In present study, 51 batches of commercial CRP samples, including four cultivars originated from Hunan, Jiangxi, Zhejiang, Hubei, Sichuan and Guangdong province in China were collected. We aim to clarify the genetic and chemical diversities between GCP and CP by combining deoxyribonucleic acid (DNA) barcoding method using mainly sequences of ITS2 and psbA-trnH, with a metabolomics approach using ultra-performance liquid chromatography coupled with quadrupole time-of-flight mass spectrometry (UPLC-QTOF-MS/MS).

## 2. Experimental

### 2.1 Plant materials

In present study, a total of 51 batches of CRP samples were purchased from herbal medicine market in southern and eastern China, including 20 batches of GCP samples from Xinhui county (Guangdong province), the genuine GCP producing area and 31 CP collected from local herb market in Sichuan, Zhejiang, Jiangxi and Hunan, Hubei province, respectively (Table S1†). The samples were authenticated by Professor Guangxiong Zhou (Pharmacy College, Jinan University, Guangzhou, China) and the voucher specimens were saved in China Academy of Chinese Medical sciences Guangdong Branch, Guangzhou.

### 2.2 Chemicals and materials

2-Mercaptoethanol, LC grade acetonitrile and methanol were purchased from Merck (Darmstadt, Germany). EDTA, NaCl, HCl were of analytical reagent and manufactured by Tianjin Damao Chemical Reagent Factory (Tianjin, China). Tris–base and Agarose were purchased from Sigma Chemical Co. (MO, Missouri, USA). 2 × Taq PCR Mix was obtained from Aidlab Biotechnologies Co., Ltd (Beijing, China). LC-MS grade formic acid was obtained from Thermo Fisher Scientific Company China Branch (Shanghai, China). Analytical grade methanol was obtained from Guangzhou Chemical Reagent (Guangzhou, China). Deionized water (18 MΩ) was purified by a Mill-Q ultrapure water system (Millipore, USA). Reference compounds nobiletin, tangeretin, 3,5,6,7,8,3′,4′-heptamethoxyflavone, rutin, eriocitrin and hesperetin were obtained from Shanghai Yuanye Co, Ltd. (Shanghai, China).

### 2.3 LC-MS analysis

#### 2.3.1 Sample preparation for LC-MS analysis

Each CRP sample was powdered by a mill, and passed through 60 mesh sieve. 0.2 g of each powdered sample was accurately weighed and extracted with 15 mL methanol in an ultrasonic bath (40 kHz, 250 W) for 30 min. After cooling at room temperature, the extracted solution was added with methanol to the original weight, and followed by centrifugation at 5000 r min^−1^ for 10 min. Then the supernatant was filtered through a 0.22 μm membrane prior to use.

The quality control (QC) sample was used for validating the reproducibility and stability of the UPLC-QTOF-MS/MS methodology. It was made up by 20 μL of each CRP sample (prepared as described previously) and the mixed solution was vortexed for 1 min, and then filtered through a 0.22 μm membrane prior to use.

#### 2.3.2 LC-MS conditions

The LC analysis was carried out on an ACQUITY™ UPLC™ H-CLASS instrument (Waters Corp., Milford, MA, USA) equipped with an automatic degasser, a quaternary pump, a column oven and an autosampler. Separation was performed on a Waters ACQUITY™ UPLC™ HSS T3 column (100 × 2.1 mm, 1.7 μm) at 30 °C. The mobile phase consisted of solvent A (water containing 0.1% formic acid) and solvent B (acetonitrile), and the elution program was optimized as follows: 0–3.5 min, 5–15% B; 3.5–6 min, 15–25% B; 6–15 min, 25–40% B; 15–18 min, 40–55% B; 18–20 min, 55–85% B; 20–22 min, 85–95% B. The flow rate was set to 0.2 mL min^−1^, with an injection volume set to 2 μL.

The MS analysis was performed on a TripleTOF™ 5600^+^ mass spectrometer (AB SCIEX, Foster City, CA, USA) equipped with a DuoSpray™ Electron Spray Ionization (ESI) source in both positive and negative modes. The ESI condition was applied with the following parameters: ion spray voltage, 4500 V in positive mode (−4500 V in negative mode); ion source temperature, 500 °C; curtain gas, 25 psi; nebulizer gas (GS1) 50 psi; heater gas (GS2), 50 psi; and declustering potential (DP), 80 V (−80 V in negative mode). The mass range was set between *m*/*z* 120–1200 in both positive and negative modes. The MS scanning program consisted of a TOF MS survey scan and MS/MS scans of eight most intense ions using the information-dependent acquisition (IDA) mode, that was set for structural analysis of potential biomarkers. The IDA collision energy (CE) was set at 35 eV in positive mode (−35 eV in negative mode), and the collision energy spread (CES) was (±) 10 eV. For metabolomics analysis, MS survey scanning was only applied in an independent MS method, in which all the collected data were only used for chemometrics analysis.

#### 2.3.3 Data processing, statistical analysis and chemical metabolite identification

The raw LC-MS data were extracted and aligned by MarkerView v1.2.1 software (AB SCIEX, Foster City, CA, USA) with default parameters setting except the binning and alignment settings were “RT window” = 0.2 min and “mass window” = 10 ppm. The data matrix were normalized by using Total Area Sums function and the normalized data were then exported into the SIMCA-P 14.1 (Umetrics, Sweden), which was used for multivariate statistical analyses including principal component analysis (PCA) and orthogonal partial least squares discriminant analysis (OPLS-DA).^19^ The data matrix was scaled using the mean centering and Pareto scaling algorithm for PCA and OPLS-DA analysis by SIMCA-P, respectively. First, unsupervised PCA was performed to obtain the overview of the relationships among the data matrix. Then, supervised orthogonal partial least-squares discriminate analysis (OPLS-DA) was used to distinguish the differences of metabolites between GCP and CP. The established model was assessed by calculating the *R*^2^ and *Q*^2^ values. A large *R*^2^ (close to 1) indicate the established model is a good model. A large *Q*^2^ (*Q*^2^ > 0.5) indicates how well the model predicts new data based on cross-validation and good predictability.^20^ Permutation test was used to validate the OPLS-DA model. Variables importance in projection (VIP) value was calculated from the OPLS-DA model. Components were selected as potential variables when the VIP value was more than 1.5. SPSS 23.0 (IBM, USA) software was used to perform Mann–Whitney *U* test for these variables. Metabolites with *p* < 0.05 and VIP value > 1.5 were considered to be statistically significant. A support vector machines (SVM) model for prediction and classification of CRP samples was conducted by the package e1071 of free R language platform.

The accurate mass determination of their precursor ions and MS/MS fragments were conducted by using the high resolution UPLC-QTOF-MS/MS. The mass error between theoretical mass value and experimental mass value was less than 5 ppm. The potential biomarkers were identified or tentatively identified by matching the molecular formulas and key fragment ions and pathways with that of standards and compounds previously identified from the same plant source.

#### 2.3.4 Validation of analytical method

In order to ensure the reliability of the analysis method, the QC sample has been used to validate reproducibility of the method and precision of UPLC-QTOF-MS/MS instrument by duplicate analysis of six injections. In addition, during the CRP detection, the QC sample was analyzed once every 10 experimental samples. Then the retention times and intensities of 10 randomly selected peaks from the chromatograms of all the QC samples were determined using PeakView software 1.2.0 and statistical analysis of relative standard deviations (RSD) was performed to validate the analytical method. The results showed that the RSD of typical ion intensities and retention time values were less than 10% and 0.2%, respectively.

### 2.4 DNA barcoding experiment

#### 2.4.1 Sample pretreatment for DNA extraction

50 mg of each dried powered CRP sample was added into a 2 mL tube and ground into fine powder by a Precellys 24 Homogenizer (Bertin Technologies Co. France). Then the powder was mixed with 800 μL pretreatment extracting buffer (containing 0.1 mol L^−1^ Tris–HCl, 0.02 mol L^−1^ EDTA, 4% (w/v) NaCl and 2% (v/v) 2-mercaptoethanol) for 5 min, further centrifuged at 10 000 rpm for 1 min, and then discarded the supernatant.

#### 2.4.2 DNA extraction, PCR, sequencing and analysis

A DP305 Plant Genomic DNA Kit (TIANGEN Biotech Company, Beijing, China) was used for genomic DNA extraction. Extracting operation steps were carried out in accordance with the specification. The extracted total DNA samples were stored in the TE buffer at −20 °C until use. Polymerase chain reactions (PCR) were performed to amplify the internal transcribed spacer 2 (ITS2),^21^ and the non-coding region between the chloroplast psbA gene and the trnH gene (psbA-trnH)

The universal primers used for the amplification of ITS2 region were:S2F: 5′-ATGCGATACTTGGTGTGAAT-3′;S3R: 5′-GACGCTTCTCCAGACTACAAT-3′.

The primers for psbA-trnH regions were:fwd PA: 5′-GTTATGCATGAACGTAATGCTC-3′;rev TF: 5′-CGCGCATGGTGGATTCACAATCC-3′.

Each reaction mixture (25 μL) contained 2 × Taq PCR Mix 12.50 μL, 1.0 μL of each 0.25 μM forward and reverse primer, 2.0 μL DNA template, and ddH_2_O 8.5 μL. The PCR protocol used to amplify ITS2 was as follows: initial denaturation at 94 °C for 5 min, 35 cycles of 94 °C for 30 s, 56 °C for 30 s, 72 °C 45 s, then final extension at 72 °C for 10 min, and hold at 4 °C. The PCR program for psbA-trnH was: 94 °C for 5 min, 30 cycles of 94 °C for 1 min, 55 °C for 1 min, 72 °C 1.5 min, and final extension at 72 °C for 7 min, before cooling down at 4 °C. PCR reactions were carried out on a ProFlexTM PCR system (Thermo fisher Scientific, USA). Amplicons were examined on 1% agarose gels electrophoresis and visualized under ultraviolet light. The PCR products with bright and single band were selected, and sent for sequencing by Majorbio Com. (Guangzhou, China). The sequences were assembled using CodonCode Aligner v6.0.2 (USA). ITS2 sequence annotations were carried out on the website (http://its2.bioapps.biozentrum.uni-wuerzburg.de/) with Hidden Markov model (HMMer) and predicted the secondary structures of ITS2. Then the sequences were analyzed using MEGA 6.0 (Molecular Evolutionary Genetics analysis v6.0). Genetic distance within interspecies was calculated with K2P model.

## 3. Results

### 3.1 Discrimination of GCP and CP based on LC-MS multivariate analysis

**Fig. 1:**
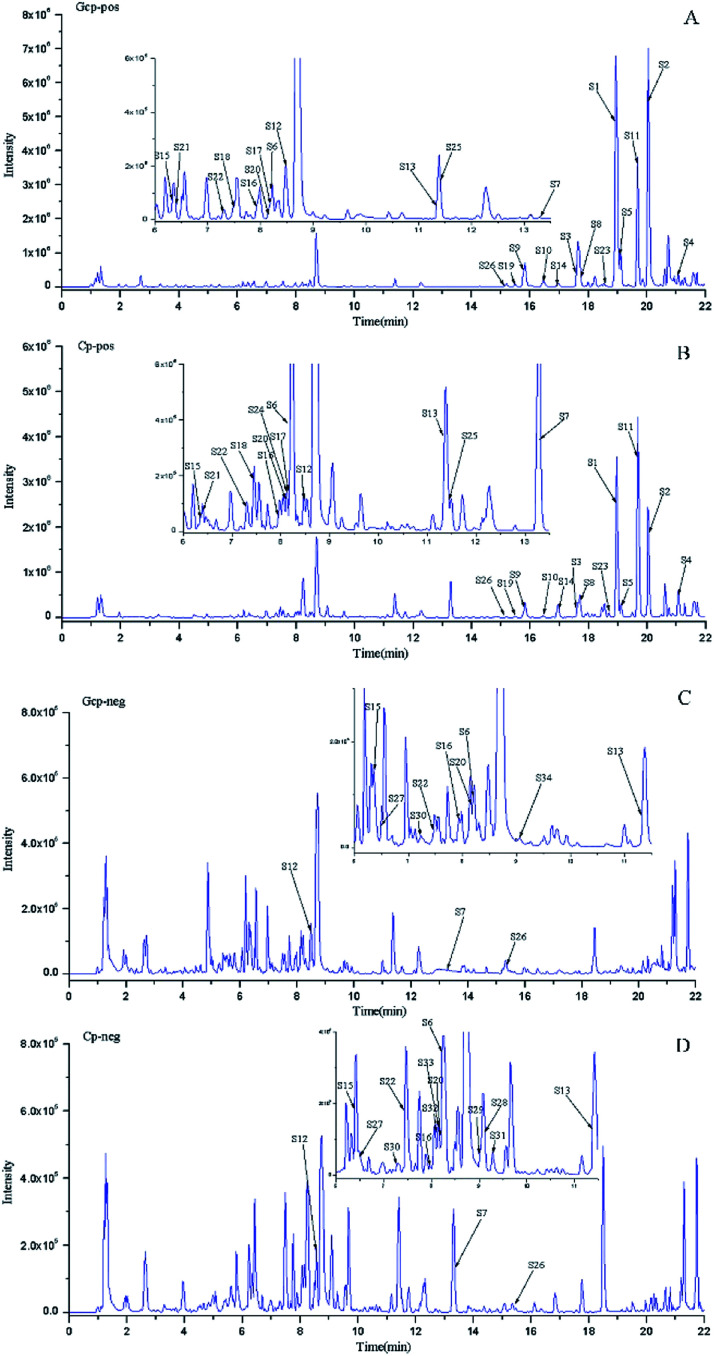
The typical base peak chromatograms (BPC) of GCP and CP samples in positive and negative modes. (A) BPC of GCP in positive MS mode; (B) BPC of CP in positive MS mode; (C) BPC of GCP in negative MS mode; and (D) BPC of CP in negative MS mode.

**Fig. 2:**
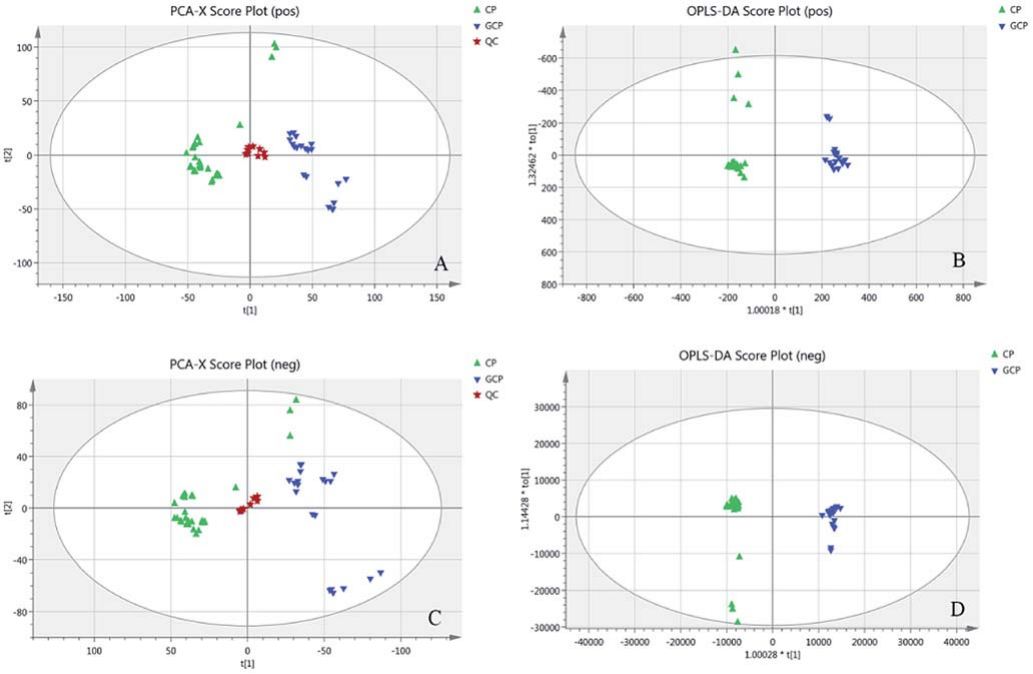
(A) PCA score plot in positive mode (R^2^X = 0.873, R^2^Q = 0.723). (B) OPLS-DA score plot in positive mode with statistical parameters (R^2^X = 0.656, R^2^Y = 0.989, *Q*^2^ = 0.974). (C) PCA score plot in negative mode (R^2^X = 0.888, R^2^Q = 0.741). (D) OPLS-DA score plot in negative mode with statistical parameters (R^2^X = 0.549, R^2^Y = 0.994, *Q*^2^ = 0.982). Blue triangles represent GCP samples, green triangle represent CP samples and pentacles represent QC samples.

The LC-MS data of 51 batches of CRP samples were obtained by an optimized UPLC-QTOF-MS/MS method and the typical base peak chromatograms (BPCs) were shown in Fig. 1. The raw data preliminarily processed by MarkerView v1.2.1 software were exported into SIMCA-P (14.1) for PCA and OPLS-DA multivariate analysis. An obvious two separated trends among GCP and CP were observed in PCA score plot both of in positive and negative ion modes (Fig. 2A and C). Principal component 1 and component 2 accounted for 30.1% and 15.1% of total variance of normalized LC-MS data in the positive modes (29.0% and 15.0% in the negative modes), respectively. In order to further identify the chemotypic variation between GCP and CP and to find potential chemical markers, a supervised OPLS-DA method was adopted. The result of OPLS-DA model derived from data both of the positive modes and negative modes analysis was displayed in Fig. 2B and D showed clear separation among the two groups. In positive mode the relevant R^2^Y = 0.989 and *Q*^2^ = 0.974 (and in negative mode the relevant R^2^Y = 0.994 and *Q*^2^ = 0.982) indicated a good predictability and goodness-of-fit of the model. The 200 iterations permutation test was applied to assess the predictability of the model (Fig. S1†).

#### 3.1.1 Selection, identification of the potential chemical markers

**Fig. 3:**
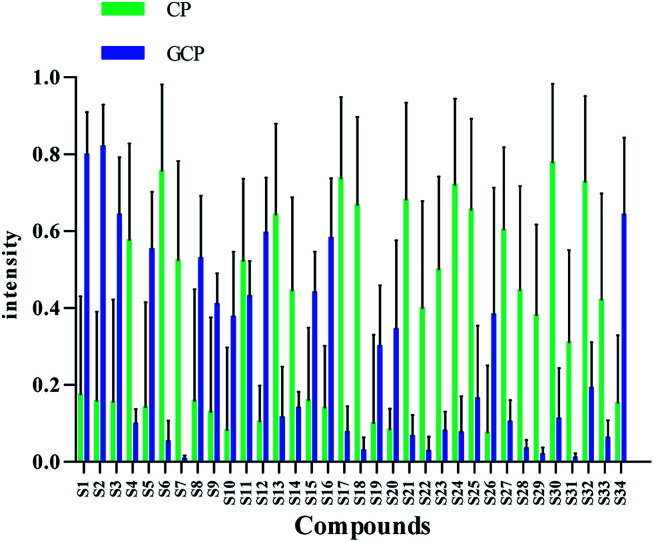
Differences of the 34 chemical markers between GCP and CP.

According to variable importance of the project (VIP), the differentiated metabolites with VIP value > 1.5 from the OPLS-DA models were selected for further Mann–Whitney *U*-test using SPSS 23.0. The metabolites that VIP > 1.5 and *p* < 0.05 were selected as the potential chemical markers (*S*-plots see Fig. S2†). Based on the MS information of standard references and compounds from previously reported literatures, the accurate mass determination of their precursor ions and MS/MS fragments were conducted by using the high resolution UPLC-QTOF-MS/MS for identification of the chemical markers. Consequently, 34 metabolites were identified and selected as chemical markers by comparison of the differences between GCP and CP (Table 1). All of the identified compounds were marked in the represented chromatogram of GCP and CP sample in positive and negative mode (Fig. 1). The mean MS intensities of 34 metabolites between GCP and CP are shown in Fig. 3, indicating their main chemical differences. The differences of chemical markers in GCP and CP by the heatmap are shown in Fig. S3.†

**Table 1:** Identification of potential chemical markers^a^

No.	Compounds	MF	Retention time	Ion mode	*m*/*z*	Mass error (ppm)	MS^2^	VIP
S1	Nobiletin*	C_21_H_22_O_8_	18.88	[M + H]^+^	403.1391	0.9	388; 387; 373; 355; 330; 327; 211; 183	24.4378
S2	Tangeretin*	C_20_H_20_O_7_	20.02	[M + H]^+^	373.1279	−0.7	358; 343; 328; 325; 300; 297; 271; 211; 183; 135	24.445
S3	Sinensetin	C_20_H_20_O_7_	17.58	[M + H]^+^	373.1284	0.6	358; 357; 343; 329; 315; 312; 297; 153; 151	11.1517
S4	Natsudaidai	C_21_H_22_O_9_	21.07	[M + H]^+^	419.1334	−0.6	404; 389; 371; 361; 343; 165	12.0638
S5	Tetramethyl-*O*-isoscutellarin	C_19_H_18_O_6_	19.06	[M + H]^+^	343.1187	3.2	327; 299; 282; 285; 267; 253; 153	9.3755
S6	Narirutin	C_27_H_32_O_14_	8.24	[M + H]^+^	581.1858	−1.2	435; 419; 401; 383; 339; 315; 273; 263; 195; 153; 129	11.4284
S7	Melitidin	C_33_H_40_O_18_	13.23	[M + H]^+^	725.2277	−1.4	419; 404; 389	10.5253
S8	Tetramethyl-*O*-scutellarin	C_19_H_18_O_6_	17.64	[M + H]^+^	343.1178	0.5	328; 313; 285; 181	8.59813
S9	Isosinensetin	C_20_H_20_O_7_	15.82	[M + H]^+^	373.1282	1.1	358; 357; 343; 315; 181; 153	6.99732
S10	Monohydroxy-pentamethoxyflavone	C_20_H_20_O_8_	16.38	[M + H]^+^	389.1232	0.3	374; 359; 313; 287	5.77288
S11	3,5,6,7,8,3′,4′-heptamethoxyflavone*	C_22_H_24_O_9_	19.61	[M + H]^+^	433.1490	−0.7	433; 418; 417; 403; 385; 342; 165	4.08285
S12	Diosmin	C_28_H_32_O_15_	8.49	[M + H]^+^	607.1679	1.7	299; 284	5.35966
S13	Didymin	C_28_H_34_O_14_	11.34	[M + H]^+^	595.2010	−1.9	449; 397; 329; 287; 263; 153	7.48591
S14	Hexamethoxyflavone	C_21_H_22_O_8_	16.95	[M + H]^+^	403.1386	−0.4	403; 388; 387; 373; 345; 327; 165	3.99101
S15	Diosmetin-6,8-di-*C*-glucoside	C_28_H_32_O_16_	6.34	[M + H]^+^	625.1750	−2.1	607; 589; 571; 487; 439; 409; 367; 355; 325; 313	3.21375
S16	Diosmetin-6-*C*-glucoside	C_22_H_22_O_11_	7.94	[M + H]^+^	463.1231	−0.8	445; 427; 367; 343; 325; 313; 301; 151	3.49067
S17	Monohydroxy-hexamethoxyflavone	C_21_H_22_O_9_	8.22	[M + H]^+^	419.1331	−1.3	401; 383; 273; 263; 245; 219; 195; 165	3.2909
S18	Rutin*	C_27_H_30_O_16_	7.51	[M + H]^+^	611.1594	−2.1	465; 303; 129	3.33815
S19	5-Hydroxy-6,7,8,3′-4′-pentamethoxyflavone	C_20_H_20_O_8_	15.49	[M + H]^+^	389.1227	−1.0	374; 359; 341; 316; 197	2.52485
S20	Isorhoifolin	C_27_H_30_O_14_	8.16	[M + H]^+^	579.1697	−2.0	433; 271	2.60807
S21	Natsudaidain-3-*O*-β-d-glucoside	C_27_H_32_O_14_	6.39	[M + H]^+^	581.1850	−2.6	419; 383; 339; 273; 263	3.37778
S22	Eriocitrin*	C_27_H_32_O_15_	7.44	[M + H]^+^	597.1801	−2.2	451; 435; 355; 289; 287; 195; 153	3.31181
S23	Pentamethoxyflavone	C_20_H_20_O_7_	18.71	[M + H]^+^	373.1279	−0.7	358; 373; 343; 315; 297; 135	2.24149
S24	Naringenin isomer	C_15_H_12_O_5_	8.22	[M + H]^+^	273.0754	−1.3	153; 147	2.4109
S25	Apigenin-8-*C*-glucoside	C_22_H_24_O_9_	11.38	[M + H]^+^	433.1482	−2.6	415; 397; 379; 353; 337; 287; 263; 245; 219; 195; 161	1.99918
S26	Hesperetin*	C_16_H_14_O_6_	15.36	[M + H]^+^	303.0861	−0.7	243; 179; 177; 153	1.96456
S27	3′,5,7-Trihydroxy-4′-methoxyflavanonol-7-*O*-rutinoside	C_28_H_34_O_16_	6.48	[M − H]^−^	625.1779	0.8	597; 317; 313; 289	4.44001
S28	5,7,4′-Trihydroxy-8,3′-dimethoxy flavonol-3-*O*-[2′′/6′′-(3-hydroxy-3-methylglutaryl)]-glucoside	C_29_H_32_O_17_	9.06	[M − H]^−^	651.1580	2.0	589; 549; 507; 345; 330; 315; 287	9.02038
S29	Limocitrol or isolimocitrol-3-*O*-[6′′-(3-hydroxy-3-methylglutaryl)]-glucoside	C_30_H_34_O_18_	9.01	[M − H]^−^	681.1684	1.7	619; 579; 537; 375; 360; 359	3.49999
S30	Apigenin-*C*-glucosyl-*O*-xyloside	C_26_H_28_O_14_	7.29	[M − H]^−^	563.1470	0.1	443; 413; 323; 313; 293	3.02874
S31	Isorhamnetin-3-*O*-[6′′-(3-hydroxy-3-methylglutaryl)]-glucoside	C_28_H_30_O_16_	9.25	[M − H]^−^	621.1470	1.4	559; 519; 477; 417; 315	3.51437
S32	Nicotiflorin	C_27_H_30_O_15_	8.09	[M − H]^−^	593.1515	0.5	285; 284; 255	2.30776
S33	8-Methoxyquercetin-3-*O*-[6′′-(3-hydroxy-3-methylglutaroyl)]-glucoside	C_28_H_30_O_17_	8.07	[M − H]^−^	637.1413	0.4	575; 535; 493; 331; 330; 316; 315	2.56254
S34	Diosmetin-7-*O*-glucoside	C_22_H_22_O_11_	8.99	[M − H]^−^	461.1087	−0.5	446; 415; 371; 341; 313; 299; 298; 297; 283; 255	1.94619

a The compound with “*” means it was compared with reference standard; the *p*-value <0.05.

**Fig. 4:**
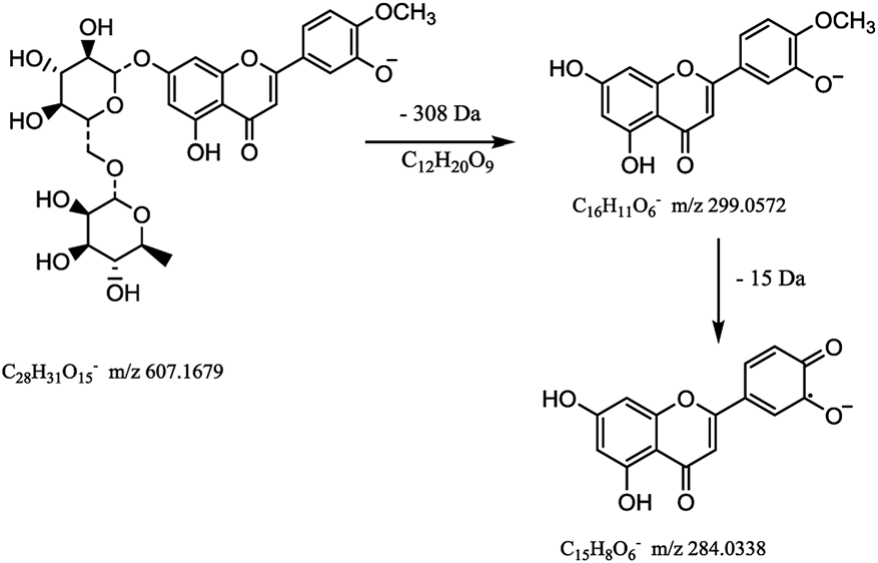
The MS fragmentation pathways of diosmin.

Flavonoids are abundant in CRP. In our present study, most of the chemical markers responsible for differentiating GCP from CP are flavonoids especially polymethoxyflavones (PMF). The typical flavonoids in CRP such as nobiletin, tangeretin, 3,5,6,7,8,3′,4′-heptamethoxyflavone, rutin, eriocitrin, hesperetin were identified by comparing the accurate mass determination and the retention time with those of reference standards. The detailed MS information of other chemical components were listed in Table 1 and they were identified based on accurate mass determination of precursor ions and their fragment peaks, which were compared with relevant literatures.^22–24^ Take S12 for example, it gave a [M − H]^−^ ion at *m*/*z* 607.1679, which produced the base fragment ion at *m*/*z* 299, which refers to the aglycone residue, as well as a characteristic odd-electron ion at *m*/*z* 284 [aglycone–·CH_2_–H]^−^ in the MS^2^ spectrum. By comparing with related literatures, it was identified as diosmin.^22^ The basic fragmentation pathways of this compound was shown on Fig. 4.

#### 3.1.2 Support vector machine (SVM)

Support vector machine is a supervised learning model with the idea of separating data and sequential minimal optimization algorithm, which is regards as a promising tool for data mining and classification. After training on set data, SVM can be used to predict the objects whose values are unknown. Previous studies had successfully employed it to predict and classify the species of Clematidis Radix et Rhizoma^25^ and for *Citrus* herbs.^24^ To demonstrate the capability of the marker compounds in classification and prediction of the CRP samples, 25 markers obtained from positive LC-MS data were selected as the inputs to build a 25-dimentional dataset for performing SVM model on the free R platform. Three vectors (GCP, CP and QC) were applied as outputs. To enhance the model accuracy and prevent data over fitting, 5-fold cross-validation was applied. 47 batches of samples (including QC samples) were randomly treated as a training set to build the model. In the SVM ( ) function, type is C-classification and kernel is radial and the two parameters is optimized as *γ* = 0.05 and *C* = 1 respectively. As showed in Table S2,† the model have an excellent predication accuracy of 100% for the training set and the otherwise test set of 13 samples. The results demonstrate that the markers show potential powerful ability of discriminating GCP from CP.

### 3.2 ITS2 sequences analysis

**Fig. 5:**
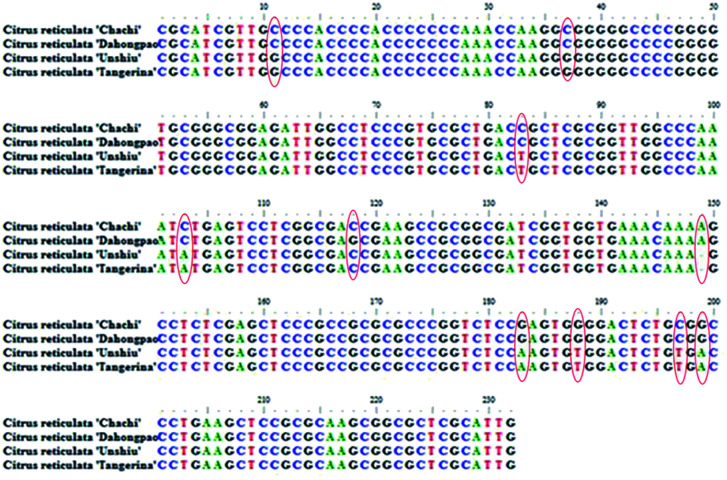
The characteristics of the dominant haplotype sequence of four species (the variation sites were framed).

According to the optimized DNA extraction protocol, the total DNA of all CRP samples were successfully extracted and the ITS2 sequences were further amplified and sequenced. The ITS2 regions were aligned and assembled by Codoncode Aligner, and then further annotated on the website (http://its2.bioapps.biozentrum.uni-wuerzburg.de/). Differences of the ITS2 sequence properties between GCP (the pericarp of *Citrus reticulata* ‘Chachi’) and CP (*Citrus reticulata* ‘Dahongpao’, *Citrus reticulata* ‘Unshiu’, and *Citrus reticulata* ‘Tangerina’ in present study) are shown in Table 2. The standard ITS2 sequence length of both *Citrus reticulata* ‘Chachi’ and *Citrus reticulata* ‘Dahongpao’ was 232 bp and the GC percentage content was 71.55%. The interspecies distance between the two cultivars was 0.001, indicating that the two genetic information in ITS2 sequences are very close. *Citrus reticulata* ‘Unshiu’ and *Citrus reticulata* ‘Tangerina’ showed higher genetic distances and lower GC content. A total of 10 variation sites were detected in 51 GCP and CP ITS2 sequences, including C–G mutations at 11, 37 and 118 sites; C–T mutations at 83 and 197 sites; C–A mutations on 103 site; G–A mutations at 183, 199 sites; G–T mutations at 188 sites, and one insertion or deletion 149 site. Most of the variation sites are consistent with previous report.^16^ The characteristics of the dominant haplotype sequences were shown on the Fig. 5.

**Table 2:** Properties of ITS2 of *Citrus reticulata*

Sample	Base number	GC (%)	Genetic distance
*Citrus reticulata* ‘Chachi’	232	71.55	—
*Citrus reticulata* ‘Dahongpao’	232	71.55	0.001
*Citrus reticulata* ‘Unshiu’	231	69.26	0.031
*Citrus reticulata* ‘Tangerina’	231	69.26	0.035

**Fig. 6:**
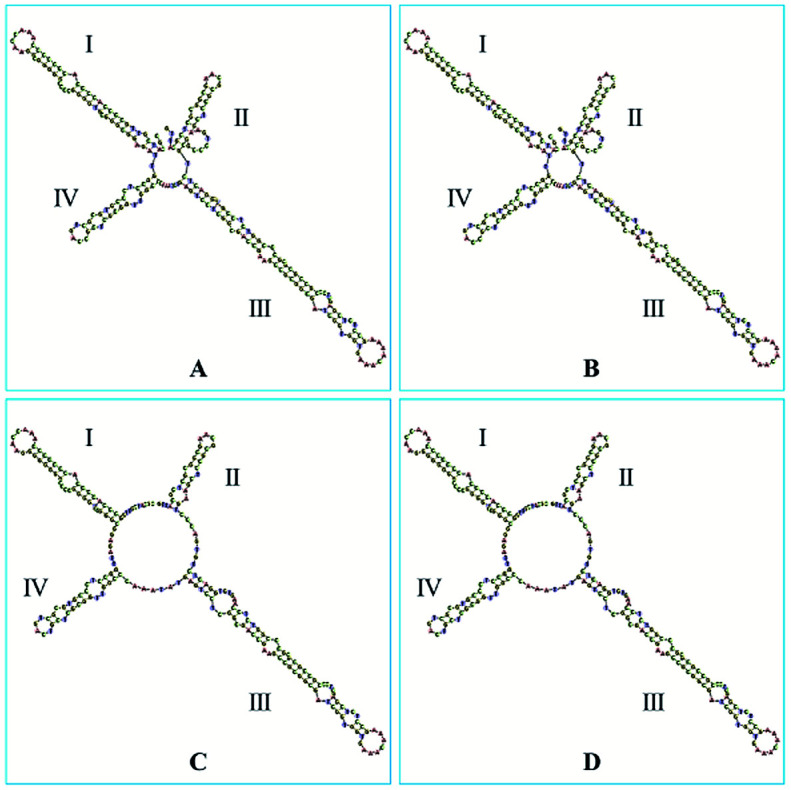
The secondary structures of: (A) *Citrus reticulata* ‘Chachi’; (B) *Citrus reticulata* ‘Dahongpao’; (C) *Citrus reticulata* ‘Unshiu’; (D) *Citrus reticulata* ‘Tangerina’.

The ITS2 secondary structures of these GCP and CP samples were predicted according to the database and website (http://its2.bioapps.biozentrum.uni-wuerzburg.de/) displayed on the Fig. 6. It was shown that the ITS2 secondary structure of *Citrus reticulata* ‘Chachi’ were significantly different. Compared with *Citrus reticulata* ‘Unshiu’ which were cultivated in Taizhou City, Zhejiang Province and *Citrus reticulata* ‘Tangerina’ from Nanchang City, Jiangxi Province, *Citrus reticulata* ‘Chachi’ has longer length in the Helix I. In the part of Helix II, there are obvious differences between *Citrus reticulata* ‘Chachi’ and the two species. As the loops structure characteristic of the Helix III, *Citrus reticulata* ‘Chachi’ has less loops than *Citrus reticulata* ‘Dahongpao’ which harvested in Zigong City of Sichuan Province, despite their rest parts of the Helix structure were similar. In conclusion, ITS2 regions and its secondary structures could provide scientific evidence for the uniqueness of GCP (the pericarp of *Citrus reticulata* ‘Chachi’), indicating that the ITS2 DNA barcoding method shows good discriminating power for distinguishing GCP from other cultivars.

### 3.3 The psbA-trnH sequences analysis

**Fig. 7:**
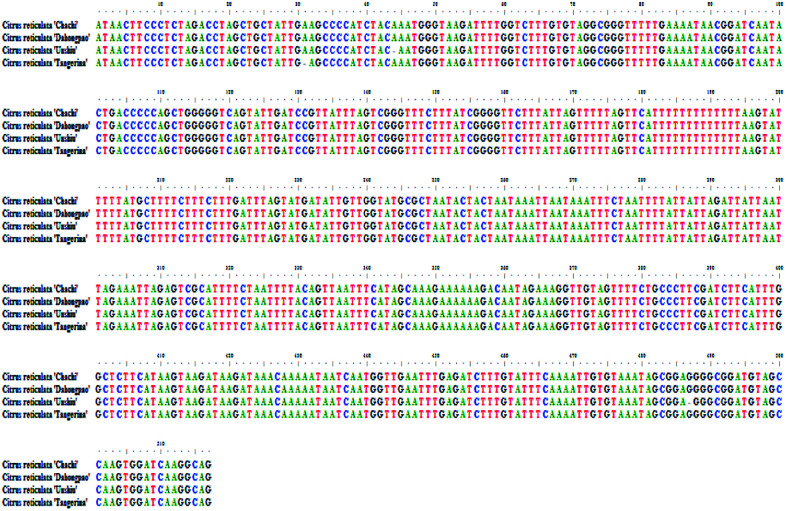
The psbA-trnH sequences of the four Species.

According the optimized DNA extraction protocol, the total DNA of all CRP samples were successfully extracted but samples with longer storage time (Gcp-17, Gcp-18, Gcp-19 and Gcp-20) has failed to amplify and sequence. The successfully sequenced psbA-trnH sequences were aligned and assembled by Codoncode Aligner. The psbA-trnH sequence properties of GCP (the pericarp of *Citrus reticulata* ‘Chachi’) contrasting with CP (the pericarp of *Citrus reticulate* Blanco, such as the cultivars of *Citrus reticulata* ‘Dahongpao’, *Citrus reticulata* ‘Unshiu’, and *Citrus reticulata* ‘Tangerina’) are shown in Fig. 7. All of the samples showed nearly same psbA-trnH sequence length and GC content, the length was approximately 517 bp and GC content was 32.11%. Although two deletion sites (44 and 487) and one deletion site (31) were observed for *Citrus reticulata* ‘Unshiu’ and ‘Tangerina’, respectively, the psbA-trnH sequence similarity between GCP and CP is close, indicating that the psbA-trnH sequence is not suitable for distinguishing GCP from CP.

## 4. Discussion and conclusions

The ripe pericarp of *Citri reticulate* ‘Chachi’ originated from Xinhui county of Guangdong province in China, exclusively listed in Chinese Pharmacopeia (2015 edition) as name Guangchenpi (GCP), is regarded as the most genuine medicinal material. Thus a valid authentication method is crucial for discriminating GCP from other CP. Recent studies had been focused on chemical profiling of different varieties of CRP based on metabolomics.^9,18,26^ It's found that both volatile constituents and flavonoids compounds could be potential biomarkers for discriminating GCP from CP. However, these metabolomics methodologies were not validated by using QC samples. In present study, a validated metabolomics method using UPLC-QTOF MS was developed to identify potential markers that could discriminate GCP from CP. Our results suggested that polymethoxyflavones (PMF) such as hexamethoxyflavone (nobiletin and natsudaidain), pentamethoxyflavone (tangeretin and sinensetin), and tetramethoxyflavone are the most influential markers (Table 1). Some studies was also found that GCP has a distinct secondary metabolite composition. Duan^27^ found that the composition of PMFs in GCP was distinct and the content of PMFs in GCP was far higher than that in other *Citrus* cultivars. Furthermore, the GCP presents unique aroma, which is attributed to the distinct compositions of volatile components.^28^ These distinctions of secondary metabolite composition might be originated from genetic nuances.

External factors such as maturity stage, storage period, processing method and storage conditions could also affect secondary metabolites profile of individual CRP sample. Studies showed that the chemical constituents of GCP in different storage periods were changed remarkably.^29,30^ Contents of flavonoids in *Citrus reticulata* ‘Chachi’ were found varied from different habitats and collecting periods.^31^ The DNA sequences and some of intrinsic genetic information therein are believed to be more stable and would not influenced by these factors. Thus a DNA barcoding molecular identification method was incorporated with LC-MS based secondary metabolites profiling to explore the genetic characteristics and chemical diversity among 51 batches of commercial CRP samples. Results showed that the ITS2 secondary structure (Fig. 5) of *Citri reticulate* ‘Chachi’ (GCP) is different with other three cultivars (‘Unshiu’, ‘Dahongpao’ and ‘Tangerina’), indicating that ITS2 is a desirable DNA barcoding for discriminating (GCP) from other CP. Previous studies also proved that ITS region is suitable for authentication of Pericarpium Citri Reticulatae and Citri Unshius Pericarpium,^32^ and ITS2 region is suitable for authentication *Citri reticulate* ‘Chachi’, *Citri reticulate* ‘Dahongpao’ and *Citri reticulate* ‘Unshiu’.^16^

In the DNA barcoding experiment, it is easy to obtain high-quality DNA sequences from fresh CRP materials, however it's more complicated for long-term stored commercial CRP samples, especially when “CRP, the older, the better” was recognized in China. Thus the operation of DNA extracting by Plant Genomic DNA kit was improved in our experiment. The pretreatment of nuclear separation buffers was beneficial to improve the DNA extraction rate for commercial GCP specimens. Our study also studied the DNA barcording of the main adulterants of CRP, such as *Citrus maxima* (Burm.) Merr and *Citrus limon* (L.) Burm, and compared their ITS2 secondary structures with GCP. It was clearly showed that the differences between GCP and related species in the ITS2 secondary spiral structures (Fig. S4†).

In conclusion, the results presented in this study showed that the chemical analysis by UPLC-QTOF-MS/MS based metabolomics to discriminate GCP and CP was accurate. Through chemical pattern recognition, the 34 chemical markers were identified for discrimination of commercial GCP and other CP. The DNA barcoding method provides the variation sites and secondary structural diversity of ITS2 to identify GCP and CP in terms of genetic feature. The advantages of the two methods are exerted in this study. Combining the chemical composition analysis based metabolomics and the ITS2 secondary structure to establish a comprehensive and accurate method for distinguishing GCP from other CP.

## Conflicts of interest

There are no conflicts to declare.

## Supplementary Material

RA-009-C9RA03740C-s001
