# Supplementary material for: Chemical and genetic discrimination of commercial Guangchenpi (Citrus reticulata ‘Chachi’) by using UPLC-QTOF-MS/MS based metabolomics and DNA barcoding approaches
Source: RSC Adv. 2019 Jul 29;9(40):23373–81. doi: 10.1039/c9ra03740c (PMC9067315; doi:10.1039/c9ra03740c)
Supplement: RA-009-C9RA03740C-s001 [file RA-009-C9RA03740C-s001.pdf]

Electronic Supplementary Material (ESI) for RSC Advances.
This journal is © The Royal Society of Chemistry 2019

Table S1 Samples used in present study

Sample	Species	Voucher ID	Harvest Years	Collection Location
Cp-1	*Citrus reticulata* 'Tangerina'	cpt1601	2016	Lukou Town, Zhuzhou, Hunan
Cp-2	*Citrus reticulata* 'Tangerina'	cpt1602	2016	Lukou Town, Zhuzhou, Hunan
Cp-3	*Citrus reticulata* 'Tangerina'	cpt1603	2016	Lukou Town, Zhuzhou, Hunan
Cp-4	*Citrus reticulata* 'Tangerina'	cpt1701	2017	Lukou Town, Zhuzhou, Hunan
Cp-5	*Citrus reticulata* 'Tangerina'	cpt1702	2017	Lukou Town, Zhuzhou, Hunan
Cp-6	*Citrus reticulata* 'Tangerina'	cpt1604	2016	Nanchang, Jiangxi
Cp-7	*Citrus reticulata* 'Tangerina'	cpt1605	2016	Nanchang, Jiangxi
Cp-8	*Citrus reticulata* 'Tangerina'	cpt1606	2016	Nanchang, Jiangxi
Cp-9	*Citrus reticulata* 'Tangerina'	cpt1607	2016	Nanchang, Jiangxi
Cp-10	*Citrus reticulata* 'Tangerina'	cpt1703	2017	Nanchang, Jiangxi
Cp-11	*Citrus reticulata* 'Tangerina'	cpt1704	2017	Nanchang, Jiangxi
Cp-12	*Citrus reticulata* 'Tangerina'	cpt1705	2017	Nanchang, Jiangxi
Cp-13	*Citrus reticulata* 'Tangerina'	cpt1706	2017	Nanchang, Jiangxi
Cp-14	*Citrus reticulata* 'Unshiu'	cpu1601	2016	Huangyan District, Taizhou Zhejiang
Cp-15	*Citrus reticulata* 'Unshiu'	cpu1602	2016	Huangyan District, Taizhou Zhejiang
Cp-16	*Citrus reticulata* 'Unshiu'	cpu1701	2017	Huangyan District, Taizhou Zhejiang
Cp-17	*Citrus reticulata* 'Unshiu'	cpu1702	2017	Huangyan District, Taizhou Zhejiang
Cp-18	*Citrus reticulata* 'Unshiu'	cpu1703	2017	Huangyan District, Taizhou Zhejiang
Cp-19	*Citrus reticulata* 'Unshiu'	cpu1704	2017	Huangyan District, Taizhou Zhejiang
Cp-20	*Citrus reticulata* 'Unshiu'	cpu1705	2017	Huangyan District, Taizhou Zhejiang
Cp-21	*Citrus reticulata* 'Unshiu'	cpu1706	2017	Huangyan District, Taizhou Zhejiang
Cp-22	*Citrus reticulata* 'Unshiu'	cpu1707	2017	Huangyan District, Taizhou Zhejiang
Cp-23	*Citrus reticulata* 'Unshiu'	cpu1708	2017	Huangyan District, Taizhou Zhejiang
Cp-24	*Citrus reticulata* 'Unshiu'	cpu1709	2017	Huangyan District, Taizhou Zhejiang
Cp-25	*Citrus reticulata* 'Unshiu'	cpu1710	2017	Huangyan District, Taizhou Zhejiang
Cp-26	*Citrus reticulata* 'Unshiu'	cpu1711	2017	Yichang, Hubei
Cp-27	*Citrus reticulata* 'Unshiu'	cpu1712	2017	Yichang, Hubei
Cp-28	*Citrus reticulata* 'Unshiu'	cpu1713	2017	Yichang, Hubei
Cp-29	*Citrus reticulata* 'Dahongpao'	cpd1601	2016	Zigong, Sichuan
Cp-30	*Citrus reticulata* 'Dahongpao'	cpd1602	2016	Zigong, Sichuan
Cp-31	*Citrus reticulata* 'Dahongpao'	cpd1701	2017	Zigong, Sichuan
Gcp-1	*Citrus reticulate* 'Chachi'	gcp1601	2016	Xinhui, Guangdong
Gcp-2	*Citrus reticulate* 'Chachi'	gcp1602	2016	Xinhui, Guangdong
Gcp-3	*Citrus reticulate* 'Chachi'	gcp1603	2016	Xinhui, Guangdong
Gcp-4	*Citrus reticulate* 'Chachi'	gcp1604	2016	Xinhui, Guangdong
Gcp-5	*Citrus reticulate* 'Chachi'	gcp1605	2016	Xinhui, Guangdong

Gcp-6	*Citrus reticulate* 'Chachi'	gcp1606	2016	Xinhui, Guangdong
Gcp-7	*Citrus reticulate* 'Chachi'	gcp1701	2017	Xinhui, Guangdong
Gcp-8	*Citrus reticulate* 'Chachi'	gcp1702	2017	Xinhui, Guangdong
Gcp-9	*Citrus reticulate* 'Chachi'	gcp1703	2017	Xinhui, Guangdong
Gcp-10	*Citrus reticulate* 'Chachi'	gcp1704	2017	Xinhui, Guangdong
Gcp-11	*Citrus reticulate* 'Chachi'	gcp1705	2017	Xinhui, Guangdong
Gcp-12	*Citrus reticulate* 'Chachi'	gcp1710	2017	Xinhui, Guangdong
Gcp-13	*Citrus reticulate* 'Chachi'	gcp1711	2017	Xinhui, Guangdong
Gcp-14	*Citrus reticulate* 'Chachi'	gcp1712	2017	Xinhui, Guangdong
Gcp-15	*Citrus reticulate* 'Chachi'	gcp1620	2016	Xinhui, Guangdong
Gcp-16	*Citrus reticulate* 'Chachi'	gcp1621	2016	Xinhui, Guangdong
Gcp-17	*Citrus reticulate* 'Chachi'	gcp1501	2015	Xinhui, Guangdong
Gcp-18	*Citrus reticulate* 'Chachi'	gcp1502	2015	Xinhui, Guangdong
Gcp-19	*Citrus reticulate* 'Chachi'	gcp1503	2015	Sihui, Guangdong
Gcp-20	*Citrus reticulate* 'Chachi'	gcp1504	2015	Sihui, Guangdong

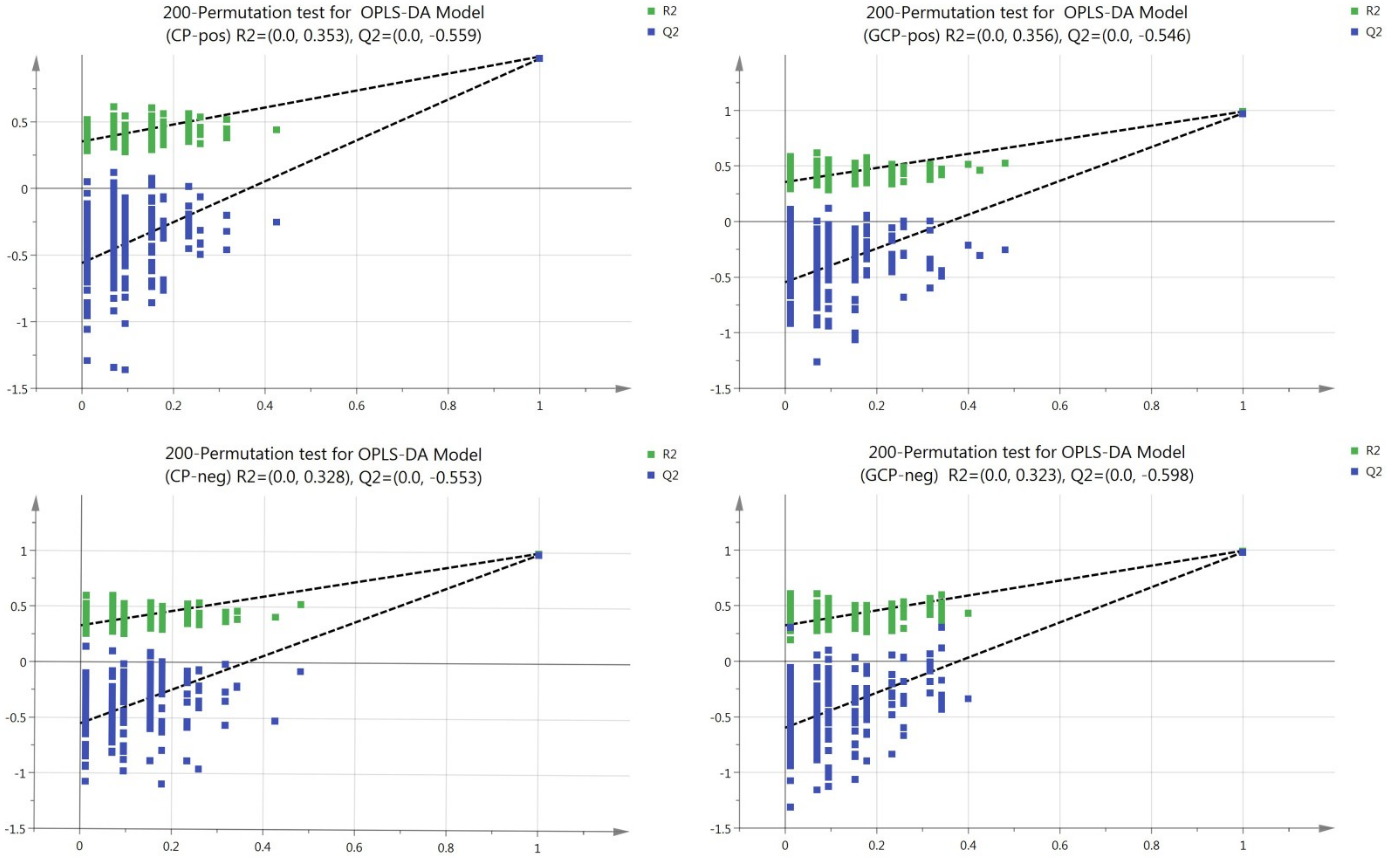

Figure S1 Permutations test plot for OPLS-DA (200 tests) in positive and negative mode.

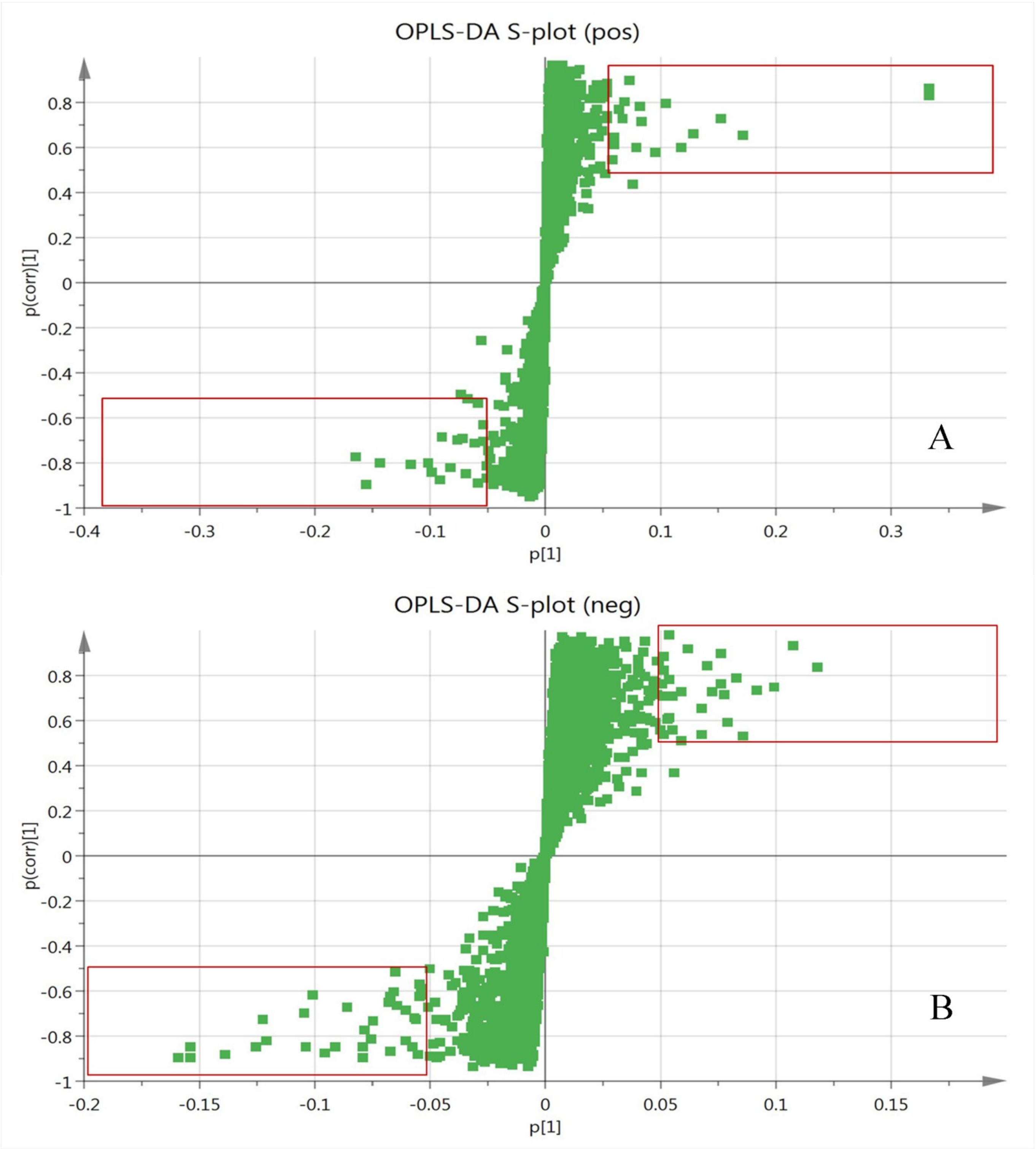

Figure S2 The S-plots of OPLS-DA both positive and negative mode.

Table S2. The classified and predicted results obtained by SVM model.

Training Set	Accuracy: 100%		Test Set	Accuracy: 100%	
**Samples**	**Actual**	**Predict**	**Samples**	**Actual**	**Predict**
Cp-27	CP	CP	Cp-26	CP	CP
Cp-28	CP	CP	Cp-5	CP	CP
Cp-1	CP	CP	Cp-8	CP	CP
Cp-2	CP	CP	Cp-11	CP	CP
Cp-3	CP	CP	Cp-22	CP	CP
Cp-4	CP	CP	Gcp-4	GCP	GCP

Cp-6	CP	CP
Cp-7	CP	CP
Cp-9	CP	CP
Cp-10	CP	CP
Cp-12	CP	CP
Cp-13	CP	CP
Cp-29	CP	CP
Cp-30	CP	CP
Cp-31	CP	CP
Gcp-19	GCP	GCP
Gcp-13	GCP	GCP
Gcp-15	GCP	GCP
Gcp-16	GCP	GCP
Gcp-18	GCP	GCP
QC-2	QC	QC
QC-3	QC	QC
QC-4	QC	QC
QC-5	QC	QC
Cp-14	CP	CP
Cp-15	CP	CP
Cp-16	CP	CP
Cp-17	CP	CP
Cp-18	CP	CP
Cp-19	CP	CP
Cp-20	CP	CP
Cp-21	CP	CP
Cp-23	CP	CP
Cp-24	CP	CP
Cp-25	CP	CP
Gcp-1	GCP	GCP
Gcp-2	GCP	GCP
Gcp-3	GCP	GCP
Gcp-6	GCP	GCP
Gcp-7	GCP	GCP
Gcp-8	GCP	GCP
Gcp-9	GCP	GCP
Gcp-10	GCP	GCP
Gcp-12	GCP	GCP
QC-6	QC	QC
QC-7	QC	QC
QC-8	QC	QC
Gcp-5	GCP	GCP
Gcp-11	GCP	GCP
Gcp-20	GCP	GCP
Gcp-14	GCP	GCP
Gcp-17	GCP	GCP
QC-1	QC	QC
QC-9	QC	QC

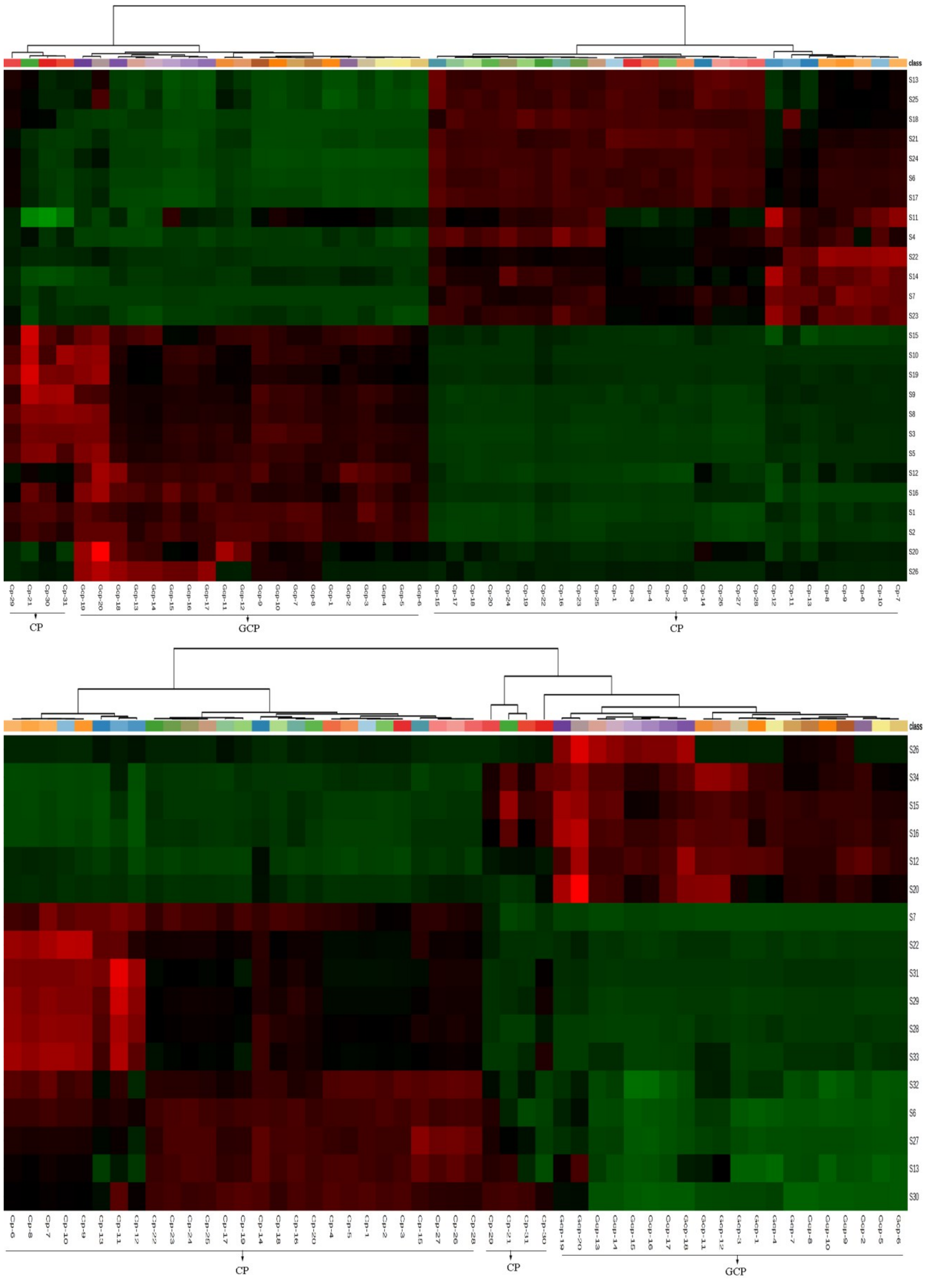

Figure S3 The heatmap of the differences of chemical markers in GCP and CP (Up: positive;Down: negative)

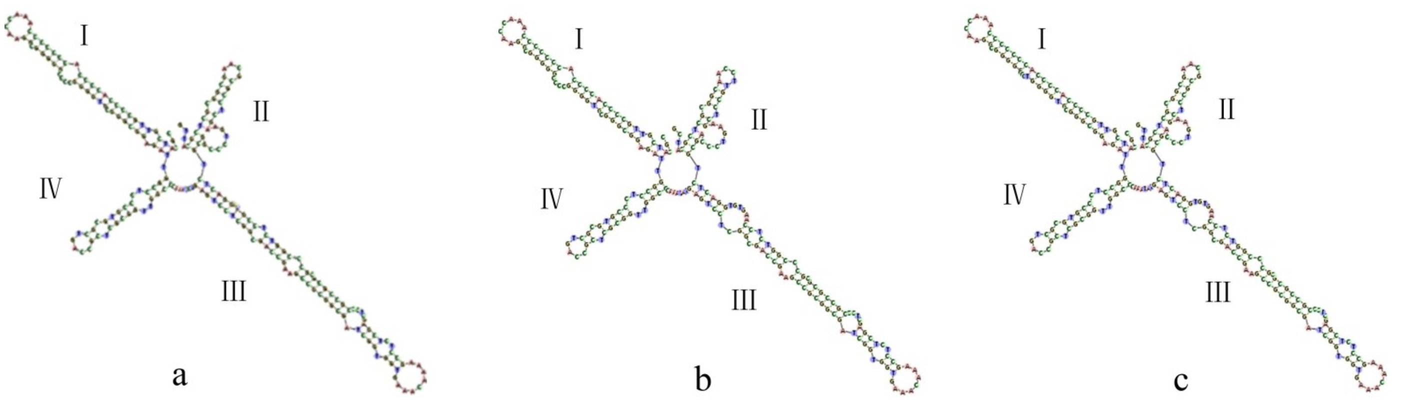

Figure S4 The secondary structures of: (a) Citrus reticulata ‘Chachi’; (b) Citrus limon (L.) Burm.; (c) Citrus maxima (Burm.) Merr
